# Capacity and Delay Estimation for Roundabouts Using Conflict Theory

**DOI:** 10.1155/2014/710938

**Published:** 2014-04-10

**Authors:** Zhaowei Qu, Yuzhou Duan, Hongyu Hu, Xianmin Song

**Affiliations:** College of Transportation, Jilin University, Changchun 130022, China

## Abstract

To estimate the capacity of roundabouts more accurately, the priority rank of each stream is determined through the classification technique given in the Highway Capacity Manual 2010 (HCM2010), which is based on macroscopical analysis of the relationship between entry flow and circulating flow. Then a conflict matrix is established using the additive conflict flow method and by considering the impacts of traffic characteristics and limited priority with high volume. Correspondingly, the conflict relationships of streams are built using probability theory. Furthermore, the entry capacity model of roundabouts is built, and sensitivity analysis is conducted on the model parameters. Finally, the entrance delay model is derived using queuing theory, and the proposed capacity model is compared with the model proposed by Wu and that in the HCM2010. The results show that the capacity calculated by the proposed model is lower than the others for an A-type roundabout, while it is basically consistent with the estimated values from HCM2010 for a B-type roundabout.

## 1. Introduction

The capacity of a transportation facility describes the maximum possible throughput under predetermined conditions, and the capacity of a roundabout provides the premise and foundations for traffic quality research at roundabout intersections. Nowadays, the capacity models of roundabouts divide into two main categories: the regression analysis model and the gap acceptance model. The former model is typically established through the regression relationship between the entry capacity and the conflict volume, relying on field data [1]. Meanwhile, the latter model is built by analyzing the entry capacity by means of gap acceptance theory [2, 3]. The former relies on large amounts of local data, leading to poor transferability [4]. Meanwhile, the latter can depict the complex relationships among traffic streams through gap acceptance theory but becomes more complicated and has worse applicability under certain traffic conditions (e.g., with limited priority or priority reversal) [5]. Brilon and Wu [5] solved the capacity at two-way-stop-controlled (TWSC) intersections via the additive conflict flow (ACF) method, which originated from conflict theory, and the results showed that the method reflects the actual traffic conditions better under high volumes. Ma et al. [6] also used this technique to analyze the capacity of T-type unsignalized intersections. Since the driving rule of “circulating vehicle priority” applies at modern roundabouts, the circulating vehicles have priority over the entering vehicles, which is similar to the priority control at major roads. Considering the structural characteristics of roundabouts, they are actually equivalent to a combination of N T-type intersections, which have priority and unidirectional control on the major road. Moreover, the T-type intersections interact with the upstream and downstream approaches and do not stand alone. Thus, combining the operating characteristics of roundabouts with the ACF method, the entry capacity of a roundabout can be determined in this paper.

In this paper, based on an analysis of the relationships of traffic flow characteristics for the two different types of roundabout and using the ACF method derived from conflict theory, the relationship of streams in a conflict zone is modeled using probability theory. Then, the entry capacity model is derived, and sensitivity analysis is conducted on the key model parameters. On this basis, the entrance delay model is built using queuing theory. In addition, the model proposed in this paper is compared with other classic models, namely, the Wu model, the recommended procedures from the Highway Capacity Manual 2010 (HCM2010), and the recommended practical model in HCM2010. This provides a preliminary reference for traffic management and control in roundabout systems.

## 2. Traffic Operations Analysis at Roundabouts

**Figure 1:**
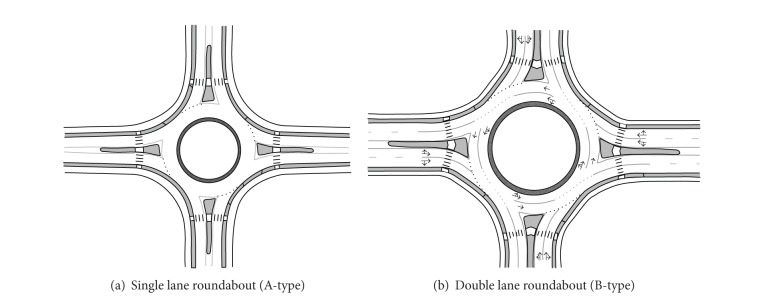
Two types of roundabout.

Differences in geometric characteristics lead to different operating characteristics on single and double lane roundabouts, leading to their capacities usually being analyzed separately [7–11]. Selecting the four-leg roundabout as the research object in this paper, the correlation between the entry flow and the circulating flow is analyzed for the single and double lane roundabouts, respectively. The two types of roundabout are defined as A-type and B-type, respectively, and the geometric diagrams are shown in Figure 1. In order to analyze the traffic streams more clearly, following the definition mode in HCM2010 [12], the entry flow is divided into three movements according to the *O*-*D* pattern (i.e., left turn, through, and right turn). Assuming that U-turn is not allowed in this study, we define some parameters as follows:

 
*q*_*K*_^*De*^ : the flow rate of *K*-turning vehicles at approach *D* (veh/h); *D* ∈ {*S*, *E*, *N*, *W*} represents either the south, east, north, or west approach; *K* ∈ {*T*, *L*, *R*} represents through, left-turning, or right-turning vehicles, respectively;
 
*q* ^*Dc*^: the flow rate of the conflict stream of approach *D* (veh/h);
 
*q*_*K*_^*e*^: the total flow rate of *K*-turning vehicles on all approaches at the roundabout (veh/h);
 
*q* ^*e*^: the total flow rate of all approaches at the roundabout (veh/h).

### 2.1. Traffic Flow Operations Analysis for an A-Type Roundabout

As per the traffic flow analysis in HCM2010, for an A-type roundabout, each entry flow corresponds to a group of conflict flows in the circulatory lane. The relationship is as follows:

(1) $$\begin{array}{c} q^{Sc}=q_{T}^{We}+q_{L}^{We}+q_{L}^{Ne}\\ q^{Ec}=q_{T}^{Se}+q_{L}^{Se}+q_{L}^{We}\\ q^{Nc}=q_{T}^{Ee}+q_{L}^{Ee}+q_{L}^{Se}\\ q^{Wc}=q_{T}^{Ne}+q_{L}^{Ne}+q_{L}^{Ee}. \end{array}$$

Summing the four equations of (1), one obtains

(2) $$\begin{array}{c} q^{Sc}+q^{Ec}+q^{Nc}+q^{Wc}=2q_{L}^{e}+q_{T}^{e}=q^{e}-q_{R}^{e}+q_{L}^{e}. \end{array}$$

It can be seen from (2) that the right-turning vehicles at the approach have no direct impact on the conflict flow in the circulatory lane. However, because the entry capacity *C*_*e*_ is a function of the conflict flow rate *q*_*c*_ (i.e., *C*_*e*_ = *f*(*q*_*c*_)) and right-turning vehicles are a part of the entry flow, they will affect the degree of saturation of the lane. Meanwhile, from (2), it is known that if *q*_*R*_^*e*^ = *q*_*L*_^*e*^, then *q* ^*c*^ = *q* ^*e*^; that is, when the total conflict flow rate is equal to the total entry flow rate, there is a balanced distribution overall. However, if *q*_*R*_^*e*^ < *q*_*L*_^*e*^, then *q* ^*c*^ > *q* ^*e*^; that is, the total conflict volume is greater than the total entry volume. The instability factors will increase gradually in this case, and the vehicle's ability for self-organization will decrease, so that minor interference may cause large fluctuations in the system.

Analyzing the properties of the traffic flow, several streams from different approaches, that is, *q*_*K*_^*De*^, constitute the corresponding conflict stream of the entrance, *q* ^*Dc*^, and there is a positive correlation between them. Meanwhile, the conflict stream, *q* ^*Dc*^, is the main factor affecting the entry capacity, and there is a negative correlation between these two aspects. An important difference between roundabouts and other unsignalized intersections is that the prior circulating flow is the temporal and spatial combination of the entry flows from different sources onto the roundabout. Thus, to some extent, on a large spatial and temporal scale, the entry flow rate will show a negative correlation to the entry capacity. However, the correlation is not united; that is, the flows from other approaches help determine the objective entry capacity. With an increase in traffic flow, the entry capacity decreases, and, when *x* ^*D*^ = *q* ^*De*^/*C* ^*De*^ ≥ 0.95, this entry approach will tend toward instability [13], where *x* ^*D*^ is the degree of saturation of approach *D*, *D* ∈ {*S*, *E*, *N*, *W*}. *q* ^*De*^ is the flow rates of approach *D*, in veh/h. *C* ^*De*^ is the capacity of approach *D*, in veh/h.

### 2.2. Traffic Flow Operations Analysis for a B-Type Roundabout

Similarly to the case of the A-type roundabout, the entry flow can be divided into three directions at a B-type roundabout. Unlike the former case, however, the B-type has two circulatory lanes, and the flow distributions of the two lanes are different. For the two entry lanes, according to the different flow attributes, it is assumed that there is a through-left lane and a through-right lane, respectively. Assuming that *p*_in_^*De*^ and *p*_out_^*De*^ are the probabilities of through vehicles entering the inner and the outer circulatory lanes, respectively, then *p*_in_^*De*^ + *p*_out_^*De*^ = 1. Then, for the entry flow rate, *q* ^*De*^, there will be (*q*_*L*_^*De*^ + *q*_*T*_^*De*^ · *p*_in_^*De*^) veh/h entering the inner lane, thus needing to cross the two streams. Meanwhile, (*q*_*R*_^*e*^ + *q*_*T*_^*e*^ · *p*_out_^*De*^) veh/h select the outer lane, and only need to pass through one stream. Because U-turn is not allowed in this paper, the relationship of the traffic flow is as follows:

(3) $$\begin{array}{c} q_{\text{in}}^{Sc}=q_{L}^{Ne}+q_{L}^{We}+q_{T}^{We}\cdot p_{\text{in}}^{We}\\ q_{\text{out}}^{Sc}=q_{T}^{We}\cdot p_{\text{out}}^{We}\\ q_{\text{in}}^{Ec}=q_{L}^{We}+q_{L}^{Se}+q_{T}^{Se}\cdot p_{\text{in}}^{Se}\\ q_{\text{out}}^{Ec}=q_{T}^{Se}\cdot p_{\text{out}}^{Se}\\ q_{\text{in}}^{Nc}=q_{L}^{Se}+q_{L}^{Ee}+q_{T}^{Ee}\cdot p_{\text{in}}^{Ee}\\ q_{\text{out}}^{Nc}=q_{T}^{Ee}\cdot p_{\text{out}}^{Ee}\\ q_{\text{in}}^{Wc}=q_{L}^{Ee}+q_{L}^{Ne}+q_{T}^{Ne}\cdot p_{\text{in}}^{Ne}\\ q_{\text{out}}^{Wc}=q_{T}^{Ne}\cdot p_{\text{out}}^{Ne}. \end{array}$$

Summing the lines of (3) applying to the inner and outer flows, respectively, one obtains the following.

For the inner flows,

(4) qinSc+qinEc+qinNc+qinWc=2qLe+qTWe·pinWe+qTSe·pinSe+qTEe·pinEe+qTNe·pinNe.

For the outer flows,

(5) qoutSc+qoutEc+qoutNc+qoutWc=qTWe·poutWe+qTSe·poutSe+qTEe·poutEe+qTNe·poutNe.

If *p*_in_^*We*^ = *p*_in_^*Se*^ = *p*_in_^*Ee*^ = *p*_in_^*Ne*^ = *p*_in_^*e*^, (4) becomes

(6) $$\begin{array}{c} q_{\text{in}}^{Sc}+q_{\text{in}}^{Ec}+q_{\text{in}}^{Nc}+q_{\text{in}}^{Wc}=2q_{L}^{e}+q_{T}^{e}\cdot p_{\text{in}}^{e}. \end{array}$$

If *p*_out_^*We*^ = *p*_out_^*Se*^ = *p*_out_^*Ee*^ = *p*_out_^*Ne*^ = *p*_out_^*e*^, (5) becomes

(7) $$\begin{array}{c} q_{\text{out}}^{Sc}+q_{\text{out}}^{Ec}+q_{\text{out}}^{Nc}+q_{\text{out}}^{Wc}=q_{T}^{e}\cdot p_{\text{out}}^{e}. \end{array}$$

In addition, combining (3), (4), and (5), one can obtain

(8) qSc+qEc+qNc+qWc=2·qLe+qTWe·pinWe+qTSe·pinSe +qTEe·pinEe+qTNe·pinNe+qTWe·poutWe +qTSe·poutSe+qTEe·poutEe+qTNe·poutNe=2qLe+qTe.

The result shown in (8) is similar to that for the A-type roundabout, but due to the vehicles in the B-type roundabout needing to pass through two streams to the inner circulating lane, there is a big difference between the operating patterns. It is difficult to directly obtain the probabilities of entry vehicles with different *O*-*D* patterns selecting the inner and outer lanes, but the flow allocations in the circulating lanes can be measured relatively easily, as it is easier to extract data automatically. Thus, an inverse selection probability for entry vehicles is adopted in this study. For the flow allocation distribution in the circulating lanes, the regression model of lane distributions proposed by Wu [14], as shown in (9), is referred to. With *q*_sum_ as the total volume of the lanes, *p*_1_ is the flow probability of lane 1, *p*_*i*_ is the flow probability of lane *i*, 1 represents the outermost lane, 2 represents the lane adjacent to the outermost lane, and so on:

(9) pi=a(1−b·e−c·qsumd)qsum−e for  i≠1p1=1−∑i=2npi for  i=1.

For B-type roundabouts, (9) becomes

(10) $$\begin{array}{c} p_{2}^{Dc}=a\left(1-b\cdot e^{-c\cdot {\left(q_{}^{Dc}\right)}^{d}}\right)\cdot {\left(q_{}^{Dc}\right)}^{-e},\\ p_{1}^{Dc}=1-p_{2}^{Dc} \end{array}$$

with *p*_1_^*Dc*^ the distribution probability of the outer circulating lane of approach *D*, *p*_2_^*Dc*^ the distribution probability of the inner circulating lane of approach *D*, and *a*, *b*, *c*, *d*, and *e* undetermined coefficients, which need to be calibrated based on field data.

Combining (10) with (3), one can obtain the lane flow distribution of the through flow in the circulating lane. That is,

(11) pinWe=p2Sc+(qLNe+qLWe)·(p2Sc−1)qTWe,poutWe=p1Sc+(qLNe+qLWe)·p1ScqTWepinSe=p2Ec+(qLWe+qLSe)·(p2Ec−1)qTSe,poutSe=p1Ec+(qLWe+qLSe)·p1EcqTSepinEe=p2Nc+(qLSe+qLEe)·(p2Nc−1)qTEe,poutEe=p1Nc+(qLSe+qLEe)·p1NcqTWepinNe=p2Wc+(qLEe+qLNe)·(p2Wc−1)qTNe,poutNe=p1Wc+(qLEe+qLNe)·p1WcqTNe.

Thus, the choice probability of the entry flow entering the inner or outer lane at a B-type roundabout is calculated, which provides the foundations for estimating the entry capacity.

## 3. Conflict Matrix and Impact Factors Analysis

### 3.1. Establishment of Conflict Matrix

According to the stream rank classification method in HCM2010, combined with the actual conditions of the roundabout, the circulating flow is set to the first priority level, and the entry flow is set to the second priority level. In addition, because of the effective isolation by the central island, there is no conflict between the streams with different areas and different priority ranks. The ACF method was proposed by Gleue and was used by Brilon and Wu [5, 15] to analyze the capacity of two-way stop-controlled (TWSC) and all-way stop-controlled (AWSC) intersections. Compared with general unsignalized intersections, roundabouts have the following characteristics. (a) The flow along the road and in the intersection is the same at a general intersection, but the flow at a roundabout is divided into the entry flow and the circulating flow due to different spatial areas, and the two flows belong to the same flow in nature, which is the organic reorganization of different flows in spatial and temporal scale. (b) There are usually flows interchanging with different multipriority ranks at ordinary intersections, but the central island of a roundabout isolates the flows effectively, so that the priority ranks at a roundabout are relatively simple. An entry flow only conflicts with some circulating flows, and assuming that there is no lane-changing in the local conflict areas, the different circulating flows can be assumed independent of each other. Miltner proposed a conflict matrix, taking into account the different traffic characteristics of roundabouts, and the object of this study, the conflict matrix of a roundabout, is different from the AWSC intersection. Taking the A-type roundabout as an example, *i* represents the entry flow and *j* represents the circulating flow, which establishes the conflict matrix. Since the entry flow with different attributes has the same priority rank on the same approach, it can take the flows from three movements as one stream, in the same way as the circulating flow. If *A*_*ij*_ = 1, it indicates that the circulating flow *j* has priority over entry flow *i*. Moreover, flows with the same priority rank are independent of each other due to the roundabout characteristics. Thus, the conflict matrix is built as shown in Table 1.

The B-type roundabout has two lanes, and the entry vehicles need to pass over different numbers of lanes due to their different destinations. However, because of the independence between the circulating flows of different lanes, the priority ranks can also be divided into two categories, and the flows with the same ranks are independent of each other. The conflict matrix can be built by referring to (3), and this will not be repeated here.

### 3.2. Consideration of Some Special Impact Factors

Some special impact factors should be considered in capacity analysis due to the geometric features and operating characteristics of roundabouts, such as exiting vehicles, heavy vehicles, and limited priority during periods of high volume; Hagring and Mereszczak [16, 17] proposed that when the width of the island is narrow or the number of exiting vehicles is excessive, downstream drivers may become hesitant, reducing the capacity of the downstream approach. Assuming that vehicles should use their indicators throughout the driving process, this measure can effectively reduce the impact of exiting vehicles. Also, the possibility of misjudgements is small and can basically be ignored. While the impact of heavy vehicles is mainly manifested in headway and service time, it can be considered in parameter calibration. Thus, the impact of limited priority on the entry vehicles is considered to a large extent in this paper.

Limited priority is defined in this study as circulating vehicles making collaborative adjustments to accommodate the crossing behavior of the entry vehicles, or priority reversal due to the entry vehicles' forced entry under certain circumstances. Troutbeck and Kako [18] proposed an adjustment factor for limited priority, assuming that the headway of a major road obeys the M3 distribution; that is,

(12) $$\begin{array}{c} C_{\lim}=\frac{1-e^{-\lambda t_{f}}}{\left[1-e^{-\lambda (t_{c}-t_{m})}-\lambda \left(t_{c}-t_{f}-t_{m}\right)e^{-\lambda (t_{c}-t_{m})}\right]}. \end{array}$$

Equation (12) shows that if one wants to obtain the adjusted coefficient *C*_lim⁡_, it is necessary to calibrate parameters such as the critical gap *t*_*c*_, the follow-up time *t*_*f*_, and the minimum headway *t*_*m*_, which is relatively complex to do. Thus, a summary calibration procedure is recommended in the actual process. (a) Investigate the circulating gap corresponding to the entry vehicles via video capture, and denote the accepted gap as *t*_*a*_. (b) Compare the accepted gap with the critical gap *t*_*c*_, and obtain the degree of limited priority *P*_lim⁡_ = *P*(*t* ≥ *t*_*c*_ | *t* ∈ *t*_*a*_). Then, the priority coefficient taking limited priority into consideration can be expressed as *A*_*ij*_′ = *A*_*ij*_ · *P*_lim⁡_ and used to update the conflict matrix.

## 4. Capacity Modeling Based on the ACF Method

### 4.1. Capacity Model Building Based on the ACF Method

Roundabouts can be seen as combinations of T-type intersections, where the circulating flow has the right of priority. According to the analysis procedure for AWSC intersections proposed by Brilon and Miltner [19], and based on conflict theory, the moving process of the entry vehicles can be seen as an optimization problem where the entry flow and the circulating flow occupy the same road resource, which is equivalent to a queuing system. Meanwhile, the conflict area occupied by the entry vehicles must meet two conditions: (a) the conflict area is not occupied by the circulating vehicles; “(b) the gap intervals between the circulating vehicles reaching the conflict zone satisfy the entry vehicles for passing through the conflict zone. Only if the two conditions are both satisfied can an entry vehicle enter the roundabout. Assuming that the average occupancy time of the conflict zone and the flow rate of the circulating flow are known, the probability that the conflict zone of entry approach *D* is occupied by the circulating flow *p*_*si*_^*Dc*^(*t*) is

(13) $$\begin{array}{c} p_{si}^{Dc}\left(t\right)=\frac{q_{i}^{Dc}t_{si}^{Dc}}{3600}, \end{array}$$

where *q*_*i*_^*Dc*^ is the flow rate of the circulating flow *i* of entrance *D*, in veh/h, and *t*_*si*_^*Dc*^ is the average time for which the circulating flow *i* occupies the conflict zone at entrance *D* (in seconds), which is similar to the average service time in a queuing system.

Then, the probability that the conflict zone of entrance *D* is not occupied by circulating flow *i*, *p*_0,*si*_^*Dc*^(*t*) is

(14) $$\begin{array}{c} p_{0,si}^{Dc}\left(t\right)=1-p_{si}^{Dc}\left(t\right). \end{array}$$

Wu divides the major road into four states and derives the state function using probability theory [20]. One can use this idea to analyze (14) from another angle. The probability that the conflict zone is not occupied by circulating flow should be composed of two parts: one is the probability of no queuing in the circulatory lane, *p*_0,*qi*_^*Dc*^(*t*), and the other is the probability of no bunch flow in the circulatory lane, *p*_0,*Bi*_^*Dc*^(*t*), so that *p*_0,*si*_^*Dc*^(*t*) can be expressed as

(15) $$\begin{array}{c} p_{0,si}^{Dc}\left(t\right)=p_{0,qi}^{Dc}\left(t\right)\cdot p_{0,Bi}^{Dc}\left(t\right)=\left(1-x_{i}^{Dc}\right)\cdot \left(1-\frac{q_{i}^{Dc}\cdot \tau_{i}}{3600}\right), \end{array}$$

with *x*_*i*_^*Dc*^ the degree of saturation of circulating flow *i* and *τ*_*i*_ the minimum headway.

Only when a large enough gap occurs between the circulating vehicles can the entry vehicles pass through the conflict zone. Define the time boundary value as *t*_*a*,*i*_, which is approximately equivalent to the critical gap. Hagring [21] proposed that the headway distribution of the circulatory lane obeys Cowan's M3 distribution, whose form is shown in the following:

(16) $$\begin{array}{c} F\left(t\right)=\{\begin{array}{cc} 0 & t<\tau \\ 1-\varphi & t=\tau \\ 1-\varphi \cdot e^{-\lambda (t-\tau )} & t>\tau , \end{array} \end{array}$$

where *φ* is the proportion of free traffic, set at *φ* = 0.75∗(1 − *q*_*i*_^*Dc*^ *τ*_*i*_) in this paper, and *λ* is the decay factor, set at *λ* = *φq*_*i*_^*Dc*^/(1 − *q*_*i*_^*Dc*^ *τ*_*i*_) as usual.

Thus, the probability of the circulating gap satisfying the conditions for entry flow to enter is

(17) $$\begin{array}{c} p_{0,ai}^{Dc}\left(t\right)=\frac{P\left(t\geq t_{a,i}\right)}{P\left(t\geq \tau_{i}\right)}=\frac{\varphi_{i}\cdot e^{-\lambda_{i}(t_{a,i}-\tau_{i})}}{1}=\varphi_{i}\cdot e^{-\lambda_{i}(t_{a,i}-\tau_{i})}. \end{array}$$

When the two conditions are both met, entry vehicles can pass through the conflict zone. Taking into account the two methods suggested in (14) and (15), the entrance probability *p*_0_^*Dc*^ is

(18) p0Dc=p0,siDc(t)·p0,aiDc(t)=(1−qiDctsiDc3600)·φi·e−λi(ta,i−τi)=(1−xiDc)·(1−qiDc·τi3600)·φi·e−λi(ta,i−τi).

Considering the limited priority at roundabouts, one can add the limited priority factor *A*_*ij*_′ into (18), obtaining

(19) p0Dc=(1−Aij′qiDctsiDc3600)·φi·e−Aij′λi(ta,i−τi)=(1−Aij′xiDc)·(1−Aij′·qiDc·τi3600)·φi·e−Aij′λi(ta,i−τi).

Assuming that the minimum service time of the entry flow is *t*_*s*,*j*_, then the maximum entry capacity is 3600/*t*_*s*,*j*_. Thus, the entry capacity of entry flow *j*(*C*_*j*_) is

(20a) Cj=Cmax⁡,j·p0Dc=3600ts,j·∏R(1−Aij′qiDctsiDc3600)·∏Rφi·e−Aij′λi(ta,i−τi)=3600ts,j·∏R(1−Aij′xiDc)·∏R(1−Aij′·qiDc·τi3600)·∏Rφi·e−Aij′λi(ta,i−τi)

with *R* the conflict set of flow *j*.

Simplifying (20a), one can obtain (20b) as follows:

(20b) Cj=3600ts,j·∏R(1−Aij′qiDctsiDc3600)·∏Rφi·e∑i=1n−Aij′λi(ta,i−τi)=3600ts,j·∏R(1−xiDc)·∏R(1−Aij′·qiDc·τi3600)·∏Rφi·e∑i=1n−Aij′λi(ta,i−τi).

Thus, we obtain the entry capacity model of entry flow *j* at the roundabout as above.

### 4.2. Capacity Model Analysis of A-Type and B-Type Roundabouts

Based on the general entry capacity model at a roundabout as in ((20a), (20b)), combined with the special characteristics of the A-type and B-type roundabouts, a model analysis is now conducted on each of the two roundabout types separately. Here, we use the probabilistic model to solve the capacity as in (14), which is the same as the first row of (20b).

#### 4.2.1. Capacity Model of the A-Type Roundabout

The entry lane and the circulating lane are both single lanes at the A-type roundabout, making the traffic conflict relatively simple. Combining (1) and (20b) with the conflict matrix shown in Table 1, one obtains the capacity of entrance *D* at a single-lane roundabout, *C*_*De*_, as follows:

(21) $$\begin{array}{c} C_{De}=\frac{3600}{t_{s,D}}\left(1-\frac{A_{ij}^{\prime }\cdot q^{Dc}t_{sD}^{Dc}}{3600}\right)\varphi_{D}e^{-A_{ij}^{\prime }\lambda_{D}(t_{sa,D}-\tau_{D})}. \end{array}$$

Since the entry lane is a single lane, *t*_*s*,*D*_ is the minimum service time of the entry flow, that is, the saturated headway of mixed traffic flow. However, this is difficult to measure, so the general method of the shared lane is applied to solve this problem in this paper. We calculate the capacity of each turning flow *C*_*DeK*_ separately and then obtain the total entry capacity, *C*_*De*_, as shown in the following:

(22) CDeK=3600ts,DK(1−Aij′·qDctsDDc3600)φDe−Aij′λD(tsa,D−τD)CDe=qLDe+qTDe+qRDexLDe+xTDe+xRDexKDe=qKDeCKDe,

where *x*_*K*_^*De*^ is the degree of saturation of* K*-turning vehicles at entrance *D*, *t*_*s*,*DK*_ is the minimum service time of* K*-turning vehicles at entrance *D*, *K* ∈ {*L*, *T*, *R*}, and *D* ∈ {*S*, *N*, *W*, *E*}, and they must satisfy

(23) $$\begin{array}{c} \left(q_{DeL}\cdot t_{DeL}\right)+\left(q_{DeT}\cdot t_{DeT}\right)+\left(q_{DeR}\cdot t_{DeR}\right)\leq 3600. \end{array}$$

According to this system, the entry capacity of each approach of the A-type roundabout can be obtained, and the total capacity of the roundabout is given by *C* = ∑_*D*_ *C*_*De*_.

#### 4.2.2. Capacity Model of the B-Type Roundabout

Because there are both two entry lanes and two circulatory lanes on a B-type roundabout, the operation pattern is relatively more complicated than for the A-type. Some of the entry vehicles will need to cross two sets of circulating traffic to arrive at the conflict zone. Combining (3), (11), and (20b), the entry capacity of entrance *D* on the B-type roundabout is

(24) CDeL=3600tsL,De·∏H∈{in,out}(1−Aij′·qHDctsHDc3600)·φin·φoute−∑H∈{in,out}ADH′λD,H(tsa,DH−τDH)CDeR=3600tsR,De·(1−Aij′·qoutDcts,outDc3600)φoute−Aij′λD,out(tsa,Dout−τDout).

In the above equation, *C*_*DeL*_ and *C*_*DeR*_ represent the capacities of the left and right lanes, respectively, *H* is the inner or outer circulatory lane, and *t*_*sL*,*De*_ and *t*_*sR*,*De*_ represent the minimum service times of the left and right lanes. Other parameters are analogous to those for the A-type roundabout.

Similarly, the two entry lanes are both shared lanes, so they need to be solved further. For through vehicles, the probability of entering a given one of the two circulatory lanes is random. Here, we assume that the through vehicles in the left lane all enter the inner circulatory lane, and the vehicles in the right lane all enter the outer lane. Thus, similar to the analysis of the A-type roundabout, the through vehicles are divided into two parts, and the flow rates are *q*_*T*_^*De*^ · *p*_in_^*De*^ and *q*_*T*_^*De*^ · *p*_out_^*De*^. Here, the capacity of the combination of left and the first part of the through vehicles is solved using the first term of (24), while the capacity of the combination of right and the second of the through vehicles is solved using the second term of (24). Thus, the lane capacity can be obtained as follows:

(25) $$\begin{array}{c} C_{DL}=\frac{q_{L}^{De}+q_{T_{\text{in}}}^{De}}{x_{L}^{De}+x_{T_{\text{in}}}^{De}},\;\;C_{DR}=\frac{q_{R}^{De}+q_{T_{\text{out}}}^{De}}{x_{R}^{De}+x_{T_{\text{out}}}^{De}}\\ C_{Se}=C_{SL}+C_{SR}, \end{array}$$

where *q*_*T*_in__^*De*^ and *q*_*T*_out__^*De*^ are the flow rates of through vehicles in the inner lane and outer lane, respectively, at approach *D*, while *x*_*T*_in__^*De*^ and *x*_*T*_out__^*De*^ are the corresponding degrees of saturation. The above equation should satisfy that (*q*_*L*_^*De*^ · *t*_*DeL*_)+(*q*_*T*_in__^*De*^ · *t*_*DeT*_in__) ≤ 3600 and (*q*_*R*_^*De*^ · *t*_*DeR*_)+(*q*_*T*_out__^*De*^ · *t*_*DeT*_out__) ≤ 3600. Summing the capacities of each approach, one can obtain the total capacity of the entire roundabout.

### 4.3. Model Parameters Analysis

**Figure 2:**
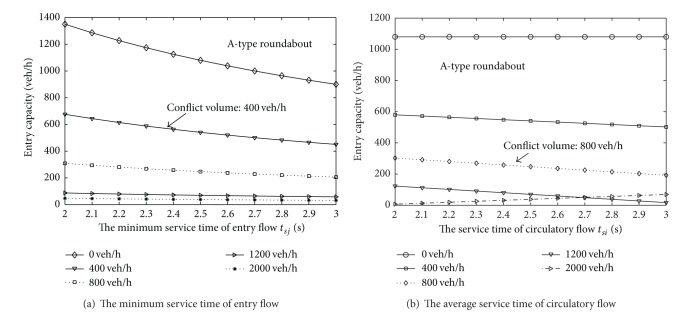
Sensitivity analysis of A-type roundabout.

**Figure 3:**
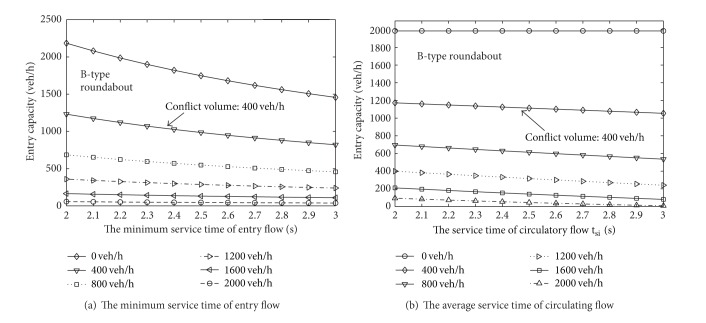
Sensitivity analysis of B-type roundabout.

For the established capacity models of the A-type and the B-type roundabouts, it can be recognized that the minimum service time of entry flow *t*_*sj*_ and the average service time of the circulatory lane are the two most important parameters, and a sensitivity analysis is thus conducted on these two values. Take a given approach, for example, we assume that the circulating flow rate gradually increases from 0 veh/h to 2000 veh/h, and the minimum headway is 2.0 s. Moreover, the time boundary value *t*_*a*,*i*_ for the A-type roundabout is 4.8 s, while the same values for the B-type roundabout are set to 4.7 s and 4.4 s for the left and right lanes, respectively [12]. The probabilities of the lane flow distribution are 0.6 and 0.4, respectively. The results are shown in Figures 2 and 3.

It can be recognized from Figure 2(a) that the entry capacity will reduce as the minimum service time of the entry flow increases. However, the decreasing trend becomes more and more apparent as the conflict volume is reduced. For example, when the conflict flow rate is 0 and 400 veh/h, the average decrease stands at 22.5 veh/h and 10.3 veh/h, respectively. However, for conflict flow rates of 1200 veh/h and 2000 veh/h, the average decrease becomes only 2.9 veh/h and 1.6 veh/h, respectively. Meanwhile, with an increase in the average service time of a circulating vehicle, the variation tendency of entry capacity is relatively gradual, especially at low circulating volumes, when the impact of the time value on the entry capacity can be ignored.

Figure 3 shows a similar trend: when the conflict volume is low, the variation in capacity is obvious as the minimum service time of the entry flow increases. Meanwhile, as the flow rate increases, because the entry capacity is already at a lower level, the overall change in value is small. On the other hand, as the average service time of the circulating flow increases, the changes in entry capacity are small for different conflict volumes, and the average value is approximately 13 veh/h.

## 5. Traffic Operations Quality Analysis at Roundabouts

### 5.1. Derivation of Delay Formula

The traffic quality at roundabouts is mainly based on the average vehicle delay and the queue length, and so forth, and the formulas for the delay and the 95th percentile queue, as recommended in HCM2010 [12], are

(26) d=3600c+900T[x−1+(x−1)2+(3600/c)x450T]+5×min⁡[x,1]Q95=900T[x−1+(1−x)2+(3600/c)x150T](c3600),

where *T* is the time period, usually 0.25 h, *d* is the average control delay (s/veh), *c* is the capacity of the subject lane (veh/h), *x* is the degree of saturation of the subject lane, and *Q*_95_ is the 95th percentile queue (veh).

The average delay and the average queue length can be discussed from the perspective of queuing theory. Assuming that the queue of the entry flow satisfies the M/G/1 queuing system, then the first queue area is the service desk, and the delay to each entry is considered, respectively. Now, the entrance to the roundabout can be seen as an AWSC intersection with only two streams. This assumes that the two streams are independent of each other in the multiple lanes, which can be regarded as a multiple parallel M/G/1 queuing system. Using the queueing theory to analyze the average delay of the entry flow, and calculating the queue delay of the queueing system by means of the classical Pollaczek-Khintchine (P-K) formula [13], gives

(27) $$\begin{array}{c} d_{ej}=\frac{x_{j}T_{j}\left(1+C_{Tj}^{2}\right)}{2\left(1-x_{j}\right)}, \end{array}$$

where *d*_*ej*_ is the average vehicle delay of entry flow *j*(*s*), *x*_*j*_ is the degree of saturation of flow *j*, which is equal to *q*_*j*_/*c*_*j*_, *T*_*j*_ is the average service time of traffic flow *j*, that is, the average time an entry vehicle spends in the first place in the queue, and *C*_*Tj*_ is the deviation coefficient of the service time of entry flow *j*, $C_{T_{j}}=\sqrt{\mathrm{Var}(T_{j})}/T_{j}$, where Var⁡(*T*_*j*_) is the variance of the service time.

If the time that the entry vehicle spends in the first position in the queue satisfies the negative exponential distribution, we have Var⁡(*T*_*j*_) = *E*(*T*_*j*_) and *C*_*T*_*j*__^2^ = 1. Considering the entry flow in terms of its separate traffic attributes, this is a single-channel queuing system, making the average service time the reciprocal of the capacity [13], and the average delay of entry flow *j*

(28) dej=deqj+Tj=1Cj(1+xj1−xj·1+CTj22)=1Cj(1+xj1−xj)=ts,j3600(1−xj)·[∏R(1−Aij′qiDctsiDc3600)·∏Rφi·e∑i=1n−Aij′λi(ta,i−τi)].

Thus, the average queue length can be obtained by Little's formula as follows:

(29) $$\begin{array}{c} L_{j}=q_{ej}*d_{ej}. \end{array}$$

Weighted by the volume on each approach at the roundabout and the average delay of the entry flow over the whole roundabout, *d*_*e*_, is

(30) $$\begin{array}{c} d_{e}=\frac{\sum_{j=1}^{n}\left(d_{ej}q_{ej}\right)}{\sum_{j=1}^{n}q_{ej}}. \end{array}$$

### 5.2. Numerical Analysis

**Figure 4:**
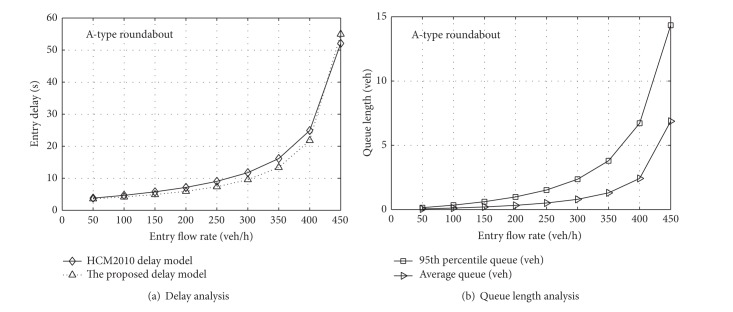
The traffic operations quality of the A-type roundabout.

**Figure 5:**
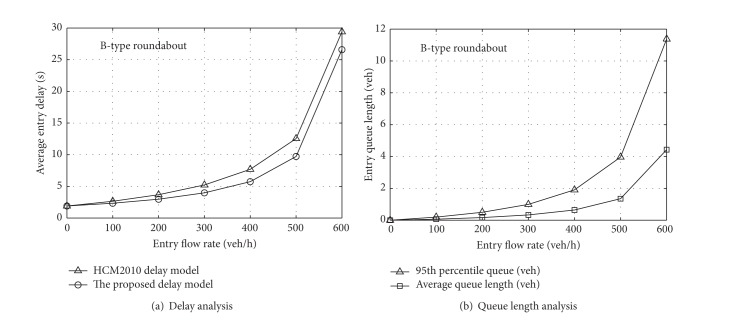
The traffic operations quality of the B-type roundabout.

To verify the effectiveness of (28) and (29), we analyze the approaches of the A-type and B-type roundabouts, respectively. Considering the recommended model of HCM2010, the traffic quality analysis can be discussed as follows. Due to the close relationship between the flow rates of the entry and circulatory flows, we assume they have the same flow rates and lane distributions at each roundabout approach and that their variation trends are also identical. In addition, we assume that there is also the same flow distribution in the circulatory lanes. From (1) and (3), it is recognized that, with an increase of entry flow, the circulating flow will increase, but the entry capacity will reduce, so that the degree of saturation will become bigger, which will lead to an increase in delay, as shown in Figures 4 and 5.

As can be seen from Figure 4(a), the average delay from the HCM2010 model is slightly larger than that derived from queuing theory, but the difference is small on the whole.  Figure 4(b) shows that, when the entry flow rate is low, the queue length is small, and its growth trend is gradual as the volume increases. However, when the flow rate exceeds 400 veh/h, the growth trend becomes steeper. For the B-type roundabout, Figure 5 shows that the difference is small between the model of HCM2010 and that proposed in this paper, but when the entry flow rate reaches 500 veh/h, the growth trend of the queue length becomes steeper. Because the result of Figures 4 and 5 is a traffic quality analysis of roundabouts under certain conditions, it should be analyzed in practice after the operating parameters have been calibrated. Moreover, (27) and (28) are derived by assuming that vehicles arrive randomly. In other words, the result is in a steady state. However, it will lose applicability when the vehicles reach saturation state, so further analysis of the saturation state needs to be conducted in future research.

## 6. Model Comparison

Wu [20] proposed a capacity model for a roundabout, which is recorded in German Highway Capacity Manual, Version 2000, as follows:

(31) $$\begin{array}{c} q_{e}=n_{e}{\left(1-\frac{\tau \cdot q_{c}}{n_{c}}\right)}^{n_{c}}\frac{1}{t_{f}}\cdot \exp\left(-q_{c}\cdot \left(t_{0}-\tau \right)\right), \end{array}$$

where *q*_*e*_ is the entry capacity, *q*_*c*_ is the conflict flow rate of the circulatory lane, *n*_*e*_ is the number of entry lanes, *n*_*c*_ is the number of circulatory lanes, and *t*_0_ = *t*_*c*_ − *t*_*f*_/2.

An estimation procedure for the capacity at a roundabout is also proposed in HCM2010 [12], as shown in (32). Through actual investigations, a group of recommended models is also provided, such as shown in (33) and (34), which represent the capacities of single-lane and double-lane roundabouts, respectively:

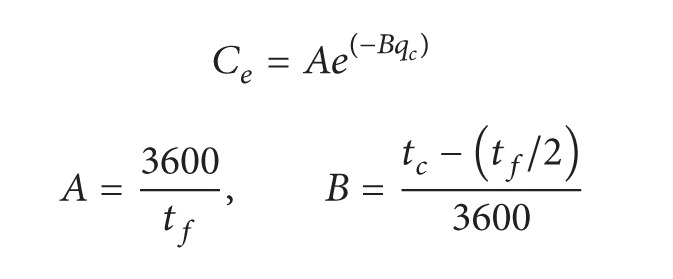
(32)

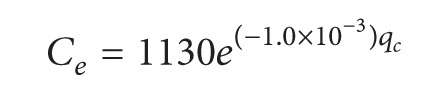
(33)

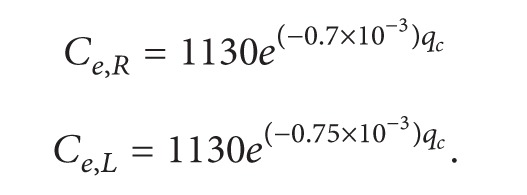
(34)


**Figure 6:**
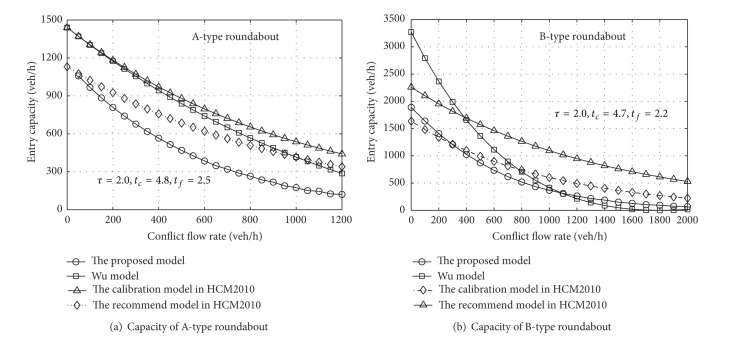
Comparison of capacity model.

A comparison is conducted between the combined entry capacity at a roundabout proposed in this paper and the above model for A-type and B-type roundabouts, respectively. Still assuming that the flow is symmetrical, we analyze the models through the relationship between entry and circulating flow. It can be recognized from Figure 6(a) that, for the A-type roundabout, the capacity value proposed in this paper is lower than those of the other three models. Because the proposed model is simultaneously affected by service time and the flow distribution of the circulating flow, while considering the limited priority at high volumes, the overall value is still smaller than that for the other three models. For the B-type roundabout shown in Figure 6(b), the estimated value proposed in this paper is close to that produced by the calibration procedure in HCM2010 from the overall trend. However, the Wu model value is higher in the early part and lower in the final part of the curves. Compared with the Wu model, the model proposed in this paper is lower at low volumes but higher when the volume exceeds a certain degree, due to the consideration of the effect of limited priority. From the analysis of Figures 6(a) and 6(b), it can be seen that the capacity produced by the proposed model is less than the model value in HCM2010.

In the above models, we adopt the recommended value in HCM2010, and for some of the proposed model parameters we use the empirical values. Thus, in further research, we need to calibrate the relevant parameters combined with the actual situation, to obtain a more realistic and accurate capacity.

## 7. Conclusion

Taking single lane (A-type) and double lane (B-type) roundabouts as the objects in this paper, based on an analysis of the quantitative relationship between the entry flow and the circulating flow, the priority ranks at roundabouts are analyzed and the conflict matrix of the roundabout is established. Then, based on the ACF method derived from conflict theory, the entry capacity model is built using probability theory. Considering the effect of a shared lane, the capacity models for the A-type and B-type roundabouts are obtained. Subsequently, the delay model and queue model of the roundabout entrances are derived using queuing theory. Then, the proposed capacity model is compared with some classic roundabout capacity models. In this paper, conflict theory is introduced into the capacity estimation procedure for roundabouts from another perspective. Also, we consider the effects of limited priority and a shared lane simultaneously. In particular, the flow distribution property in the double circulatory lanes is taken into account in this study, which provides another view of capacity estimation for roundabouts. However some of the model parameters need to be calibrated using actual data, and the practicability of the proposed model still needs to be verified. Also, some impact factors, such as heavy vehicles, pedestrians, and nonmotorized vehicles, are not considered in this paper and will be studied in further research.

## Figures and Tables

**Table 1:** Conflict matrix of an A-type roundabout.

*A*_*ij*_	Conflict flow			
	*q* ^*Sc*^	*q* ^*Ec*^	*q* ^*Nc*^	*q* ^*Wc*^
Entry flow				
*q* ^*Se*^	1	—	—	—
*q* ^*Ee*^	—	1	—	—
*q* ^*Ne*^	—	—	1	—
*q* ^*We*^	—	—	—	1
